# Disease Spectrum and Predictors of Prolonged Hospitalization in Lebanese Children With COVID-19

**DOI:** 10.7759/cureus.96745

**Published:** 2025-11-13

**Authors:** Hadi Fakih, Ibrahim Dia, Mohamad Jawad H Akach

**Affiliations:** 1 Pediatrics, Faculty of Medical Sciences, Lebanese University, Beirut, LBN; 2 Pediatric Critical Care, Sheikh Ragheb Harb University Hospital, Nabatieh, LBN; 3 Urology, Faculty of Medical Sciences, Lebanese University, Beirut, LBN; 4 Anesthesiology, Faculty of Medical Sciences, Lebanese University, Beirut, LBN

**Keywords:** covid-19, hospitalization, lebanon, length of stay, mis-c, pediatrics

## Abstract

Background

While pediatric COVID-19 is typically mild, severe outcomes such as multisystem inflammatory syndrome in children (MIS-C) can occur. Data on hospitalized children in Lebanon are scarce. This study aimed to describe the clinical features of pediatric COVID-19 hospitalizations and identify factors associated with prolonged length of stay (LOS).

Methodology

A retrospective, observational study was conducted at Sheikh Ragheb Harb University Hospital among 127 patients aged <18 years hospitalized with polymerase chain reaction-confirmed COVID-19 between January and December 2021. Data on demographics, clinical presentation, laboratory/radiological findings, treatment, and outcomes were collected from medical records. Bivariate and multivariable analyses were performed to identify factors associated with LOS.

Results

The median age was 0.6 years (interquartile range (IQR) = 0.25-1.90), and 84 (66.1%) were male. Fever, 91 (71.7%), and cough, 66 (52.0%), were the most common symptoms. Intensive care unit (ICU) admission and oxygen therapy were required in five (3.9%) cases. Overall, one (0.8%) patient developed MIS-C. There was no mortality. The median LOS was three days (IQR = 2-4). On bivariate analysis, consolidation on imaging, oxygen requirement, ICU admission, and use of antibiotics and steroids were significantly associated with longer LOS (p < 0.001 for all). On multivariable analysis, steroid use (B = 2.94, p = 0.001), pulmonary consolidation (B = 2.53, p = 0.003), and antibiotic use (B = 1.66, p = 0.035) remained independent predictors.

Conclusions

Most hospitalized children with COVID-19 had mild disease. Radiological evidence of pneumonia and the need for anti-inflammatory or antimicrobial therapy were key drivers of extended hospitalization, highlighting targets for early intervention and resource planning. These findings can assist clinicians in risk stratification and optimizing bed capacity in similar resource-limited settings.

## Introduction

The clinical spectrum of SARS-CoV-2 infection in children ranges from asymptomatic to critical illness, including multisystem inflammatory syndrome in children (MIS-C) [1]. Although severe disease is less common in children than in adults, hospitalization rates are significant, particularly among infants and adolescents with comorbidities [2,3]. The global burden of pediatric COVID-19 hospitalizations has placed a strain on healthcare systems, underscoring the need for data-driven management and resource planning.

Existing literature from North America and Europe has detailed the pediatric clinical course of COVID-19 [4,5]. However, data from the Middle East, and Lebanon specifically, remain limited. Understanding local epidemiology, clinical presentation, and resource utilization is crucial for optimizing clinical management and public health strategies, especially in countries facing complex economic and healthcare challenges. This study aims to describe the clinical characteristics and outcomes of children hospitalized with COVID-19 at a tertiary care center in Lebanon and to identify factors predictive of a prolonged hospital stay.

## Materials and methods

Study design and population

A retrospective, single-center, observational study was conducted at Sheikh Ragheb Harb University Hospital, a tertiary care center in South Lebanon. We included 127 pediatric patients (aged <18 years) hospitalized with a polymerase chain reaction (PCR)-confirmed SARS-CoV-2 infection between January and December 2021. Patients presenting for unrelated reasons who had an incidental positive PCR were excluded.

Data collection

Data were extracted from electronic medical records using a standardized data collection form. Collected variables included demographics, vital signs at presentation, symptoms, past medical history, vaccination status, laboratory results (including C-reactive protein (CRP) and procalcitonin), imaging findings (chest X-ray), treatment modalities (oxygen support, antibiotics, steroids, antivirals), and outcomes (intensive care unit (ICU) admission, length of stay (LOS), complications, mortality). Pulmonary consolidation was determined by reviewing radiology reports.

Statistical analysis

Data were analyzed using SPSS version 26.0 (IBM Corp., Armonk, NY, USA). Categorical variables were described as frequencies and percentages, and continuous variables as medians with interquartile ranges (IQRs) or means with standard deviations (SDs), as appropriate based on distribution. The Mann-Whitney U test and Spearman’s correlation were used for bivariate analysis of factors associated with LOS. A multiple linear regression model (enter method) was developed to identify independent predictors of LOS, adjusting for clinically relevant variables. A p-value <0.05 was considered statistically significant.

Ethical considerations

The study was approved by the Institutional Review Board of Sheikh Ragheb Harb University Hospital (approval number: 3279/1/22). The requirement for informed consent was waived due to the retrospective nature of the study. Patient confidentiality was maintained by using anonymized study codes throughout data analysis.

## Results

Demographic and clinical characteristics

The cohort was predominantly male, 84 (66.1%), and infant-aged (median age = 0.6 years). Most patients, 122 (96.1%), had no significant past medical history. Only 28 (22.0%) had completed their routine childhood vaccination schedule, and none had received a COVID-19 vaccine. The most common presenting symptoms were fever, 91 (71.7%), and cough, 66 (52.0%) (Table 1).

**Table 1:** Demographic and clinical characteristics of hospitalized pediatric COVID-19 patients (N = 127). IQR = interquartile range

Characteristic	Value
Age (years), median (IQR)	0.6 (0.25–1.90)
Male sex, n (%)	84 (66.1%)
Weight (kg), median (IQR)	8.0 (5.35–11.75)
Any comorbidity, n (%)	5 (3.9%)
Complete routine vaccinations, n (%)	28 (22.0%)
Symptoms, n (%)	
Fever	91 (71.7%)
Cough	66 (52.0%)
Diarrhea	36 (28.3%)
Vomiting	26 (20.5%)
Shortness of breath	15 (11.8%)

Treatment and outcomes

The majority of patients, 122 (96.1%), were managed in the general ward. Overall, five (3.9%) patients required ICU admission and oxygen support. Antibiotics and systemic steroids were administered to 63 (49.6%) and 35 (27.6%) patients, respectively. The median overall LOS was three days (IQR = 2-4). One (0.8%) case of MIS-C was identified in a neonate who required a 28-day hospitalization. There were no deaths (Table 2).

**Table 2:** Treatment and clinical outcomes (N = 127). ICU = intensive care unit; MIS-C = multisystem inflammatory syndrome in children

Outcome	n (%) or median (IQR)
ICU admission	5 (3.9%)
Oxygen requirement	5 (3.9%)
Antibiotic use	63 (49.6%)
Steroid use	35 (27.6%)
MIS-C	1 (0.8%)
Mortality	0 (0.0%)
Length of stay (days)	3 (2–4)

Factors associated with length of stay

Bivariate analysis identified several factors associated with a longer LOS, including the presence of pulmonary consolidation on imaging, oxygen requirement, ICU admission, and treatment with antibiotics or steroids (all p < 0.001). In the multiple linear regression model, steroid use (B = 2.94, 95% confidence interval (CI) 1.20-4.69, p = 0.001), pulmonary consolidation (B = 2.53, 95% CI = 0.89-4.17, p = 0.003), and antibiotic use (B = 1.66, 95% CI = 0.12-3.19, p = 0.035) remained independently associated with a prolonged hospitalization after adjusting for weight, vaccination status, CRP, and shortness of breath (Table 3).

**Table 3:** Multivariable linear regression analysis of factors associated with length of hospital stay. *: statistically significant (p < 0.05). The model was adjusted for weight, vaccination status, CRP, and shortness of breath. CI = confidence interval; CRP = C-reactive protein

Factor	Unstandardized coefficient (B)	95% CI for B	P-value
Steroid use	2.94	1.20–4.69	0.001*
Pulmonary consolidation	2.53	0.89–4.17	0.003*
Antibiotic use	1.66	0.12–3.19	0.035*

## Discussion

This study provides the first detailed description of clinical outcomes and predictors of prolonged hospitalization for children with COVID-19 in Lebanon. Our findings align with global data showing that most pediatric hospitalizations are brief and outcomes are excellent, with low rates of ICU admission and mortality [4-6]. The low prevalence of MIS-C (0.8%) and zero mortality rate are reassuring and consistent with international cohorts that report MIS-C incidences below 1% and very low fatality rates [7].

The key finding of this analysis is the identification of three independent predictors of longer LOS: pulmonary consolidation on imaging and the use of steroids and antibiotics. These findings are consistent with studies from other regions. For instance, similar to our results, a large European cohort study identified pulmonary infiltrates as a key marker of disease severity and longer hospitalization [3]. Other studies have also confirmed that radiological evidence of pneumonia is a strong predictor of extended stay in children with COVID-19 [8,9]. Our results expand upon this by quantifying the impact of consolidation on LOS in a younger, Lebanese cohort.

The use of steroids and antibiotics as predictors aligns with their role as proxies for disease severity. Steroid use, often guided by protocols for significant systemic inflammation or MIS-C [10], was the strongest predictor in our model. This aligns with studies of MIS-C and severe pneumonia, where immunomodulatory therapy is a cornerstone of management and is associated with extended care needs [1,7]. The association between steroid use and longer LOS has been documented in other pediatric severe respiratory infections, reinforcing its role as a marker of severe disease [11]. Antibiotic use, likely reflecting concern for bacterial co-infection despite a low rate of confirmed cases, was also a significant predictor. This suggests that clinical suspicion alone, common in pediatric practice, can significantly impact LOS, highlighting an area for antimicrobial stewardship improvement, a finding echoed in pediatric COVID-19 studies worldwide [12,13]. The high rate of empirical antibiotic use observed in our study is a known phenomenon in pediatric respiratory infections and underscores the need for robust diagnostic protocols to guide therapy [14].

The predominance of infants in our cohort (median age = 0.6 years) contrasts with studies from North America and Europe that often report higher hospitalization rates in adolescents [2,3]. This may reflect a uniquely lower local transmission pattern among older children or, more likely, a lower clinical threshold for admitting febrile infants during the pandemic, a practice noted in other settings [15]. This age distribution is similar to reports from some Asian countries, suggesting regional variations in the epidemiology of pediatric COVID-19 [16].

This study has limitations inherent to its single-center, retrospective design. The small sample size, particularly for severe outcomes, limits the generalizability of the findings. The high rate of empirical antibiotic and steroid use may reflect local practice patterns rather than strict evidence-based guidelines. Data on viral co-infections or long-term sequelae were not available.

## Conclusions

In this Lebanese cohort, hospitalized children with COVID-19 primarily had mild, self-limiting disease. The need for prolonged hospitalization was strongly predicted by radiological evidence of pneumonia and the administration of steroids or antibiotics. These findings can help clinicians identify high-risk children early, optimize resource allocation and bed capacity planning, and reinforce antimicrobial stewardship efforts in the management of pediatric COVID-19. Future multi-center prospective studies are warranted to further validate these predictors and develop evidence-based admission and treatment guidelines tailored to the region.
